# Bilayer ventilated labyrinthine metasurfaces with high sound absorption and tunable bandwidth

**DOI:** 10.1038/s41598-021-84986-0

**Published:** 2021-03-12

**Authors:** Jiayuan Du, Yuezhou Luo, Xinyu Zhao, Xiaodong Sun, Yanan Song, Xinhua Hu

**Affiliations:** Department of Materials Science, Key Laboratory of Micro- and Nano-Photonic Structures (Ministry of Education), and Laboratory of Advanced Materials, Fudan University, Shanghai, 200433 China

**Keywords:** Acoustics, Materials science, Theory and computation

## Abstract

The recent advent of acoustic metamaterials offers unprecedented opportunities for sound controlling in various occasions, whereas it remains a challenge to attain broadband high sound absorption and free air flow simultaneously. Here, we demonstrated, both theoretically and experimentally, that this problem can be overcome by using a bilayer ventilated labyrinthine metasurface. By altering the spacing between two constituent single-layer metasurfaces and adopting asymmetric losses in them, near-perfect (98.6%) absorption is achieved at resonant frequency for sound waves incident from the front. The relative bandwidth of absorption peak can be tuned in a wide range (from 12% to 80%) by adjusting the open area ratio of the structure. For sound waves from the back, the bilayer metasurface still serves as a sound barrier with low transmission. Our results present a strategy to realize high sound absorption and free air flow simultaneously, and could find applications in building acoustics and noise remediation.

Acoustic metamaterials are artificial engineered periodic structures with exotic acoustic properties unreachable by natural materials, which greatly increase our ability for acoustic wave controlling. Since the first demonstration of acoustic metamaterials in 2000 by Liu et al.^1^, acoustic metamaterials have been intensively investigated^2^ and applied to a wide range of fields such as acoustic wave manipulation^3^, asymmetric sound wave propagation^4^, acoustic energy harvesting^5^, and topological acoustics^6,7^.

Recently, inspired by the rapid progress in the field of two-dimensional (2D) materials, acoustic metasurfaces, namely single layers of 2D acoustic metamaterials, have received increasing attention^8–12^. Via precisely tailoring the geometry of unit cells, acoustic metasurfaces can manipulate the propagation of sound waves in a unique way, enabling fascinating applications^8–12^. A famous example is perfect sound absorbers based on acoustic metasurfaces^13–24^. By combining damping resonators with a back reflector, such metasurfaces can completely absorb sound waves with wavelengths much longer than their thicknesses^13–24^. Such high performance of absorption cannot be realized in conventional porous media with the same thickness. However, due to the employment of back reflectors without holes, the perfect sound absorbers cannot be applied in some occasions which require sound elimination and ventilation simultaneously. To overcome this problem, various types of metasurfaces with ventilation openings have been investigated^25–38^. It is found that when ventilation ducts exist in the metasurfaces, sound barriers with high reflection and low transmission can be realized^25–31^. Near-perfect sound absorption can be achieved in metasurfaces with a small open area ratio^32–36^. However, high absorption is still hard to achieve especially when the metasurfaces have a large open area ratio^37,38^. In addition, the bandwidths of absorption peaks are usually narrow and difficult to adjust.

In this paper, we demonstrate that the above problem can be overcome by employing a bilayer structure with two ventilated labyrinthine metasurfaces. The unit cell of each metasurface contains a ventilation duct as well as a curled, one-end-closed channel or a labyrinthine resonator. Compared with other resonators such as Helmholtz resonators^37,38^, the labyrinthine resonator can possess a much wider resonant peak by using a moderate folding number (such as $N=2$). Moreover, its absorption loss can be flexibly adjusted by placing porous media inside the curled channels. It is found that when the spacing between the two metasurfaces and their sound losses are appropriately chosen, perfect sound absorption can be achieved at resonant frequencies. The measured peak absorption can be as high as 98.6% at a large opening ratio (50%). By varying the open area ratio of the bilayer metasurface, the relative bandwidth of absorption peak can be tuned in a wide range (from 12% to 80%).

In the bilayer metasurface, the front single-layer metasurface serves as an absorber with a high loss while the back metasurface acts as a reflector with a low loss. Hence, high absorption occurs for sound waves incident from the front. For incidence from the back, the absorption decreases but low (and symmetric) transmission remains, so that the bilayer metasurface still serves as a sound barrier. Similar effects occur in perfect sound absorbers without ventilation openings which completely reflect sounds from the back^13–24^. Asymmetric sound absorption has also been observed in certain asymmetric acoustic waveguides^39–42^. However, since resonators are placed outside the waveguides, those designs cannot be directly applied to construct metasurfaces.

## Results

### Structures

**Figure 1:**
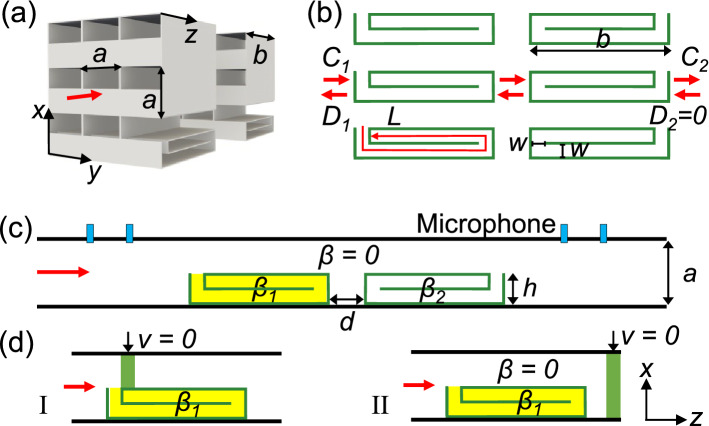
Schematic of the bilayer ventilated labyrinthine metasurface and experimental setup. (**a**) 3D and (**b**) side views of the bilayer labyrinthine metasurface that is immersed in air and impinged by a sound plane wave from the left. To illustrate the inner structure, two side plates are removed in (**a**). (**c**) Schematic side view of the experimental setup for acoustic absorption measurements. Here, a unit cell of the bilayer metasurface, consisting of two curled channels with sound loss $\beta _{1}$ and $\beta _{2}$ inside, is placed in an acoustic impedance tube. (**d**) Simplified models for cases with perfect absorption (I) and incomplete absorption (II).

The designed structure consists of two ventilated labyrinthine metasurfaces with a spacing *d*, as shown in Figs. 1a,b. Both metasurfaces are made of rigid body and immersed in air. The building block of each metasurface has a size *a* in both the *x* and *y* directions, and a thickness *b* in the *z* direction. Inside the building block, there exist a ventilation duct along the *z* direction and a one-end-closed channel curled in the *x*-*z* plane. The ventilation duct has a rectangular cross section with area of $S_{o}=\left( a-t\right) \left( a-h\right)$ with *t* being the wall thickness. The curled channel has an effective length $L\approx Nb$, a width $w=\left( h-Nt-t\right) /N$, a folding number $N=2$, a rectangular cross section with area of $S_{c}=\left( a-t\right) w$, and an aperture near the surfaces of the bilayer metasurface. Such a curled channel constitutes a labyrinthine resonator with a fundamental resonant frequency

1 $$\begin{aligned} f_{R}=c/\lambda _{R} \end{aligned}$$

where *c* is the sound speed in air and $\lambda _{R}\approx 4L$ is the fundamental resonant wavelength.

**Figure 2:**
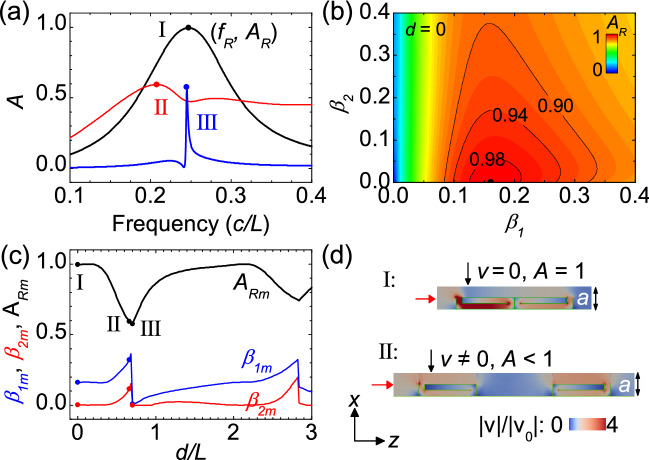
Demonstration of perfect sound absorption in bilayer labyrinthine metasurfaces with open area ratio $p_{o}=0.5$. (**a**) Calculated sound absorption spectra for structure I with $d=0,$ $\beta _{1}=0.161,$ and $\beta _{2}=0$ (black curve), structure II with $d=0.66L$, $\beta _{1}=0.321$, and $\beta _{2}=0.115$ (red curve), and structure III with $d=0.70L$, $\beta _{1}=0.0073$, and $\beta _{2}=0$ (blue curve). The absorption approaches a maximum $A_{Rm}$ at resonant frequency $f_{R}$. (**b**) $A_{R}$ as a function of $\beta _{1}$ and $\beta _{2}$ for bilayer metasurfaces with $d=0$. $A_{R}$ reaches a maximum $A_{Rm}$ at optimal losses ($\beta _{1}=\beta _{1m}$, $\beta _{2}=\beta _{2m}$). (**c**) $\beta _{1m}$, $\beta _{2m}$, and $A_{Rm}$ as a function of layer spacing *d*. (**d**) Simulated distributions of $\left| v\right| /\left| v_{0}\right|$ in the *x*-*z* plane for structures I and II at $f_{R}$ in (**a**). *v* represents particle velocity and $v_{0}$ is the particle velocity of the incident sound wave.

**Figure 3:**
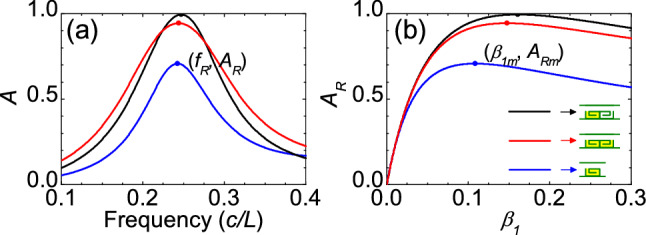
Comparison of sound absorption in three different metasurfaces. (**a**) Calculated sound absorption spectra for a bilayer metasurface with $\beta _{1}=0.161$, $\beta _{2}=0$ and $d=0$ (black line), a bilayer metasurface with $\beta _{1}=\beta _{2}=0.148$ and $d=0$ (red line), and a single-layer metasurface with $\beta _{1}=0.108$ (blue line). All the metasurfaces have an open area ratio $p_{o}=0.5$. The absorption approaches a maximum $A_{R}$ at resonant frequency $f_{R}$. (**b**) $A_{R}$ as a function of $\beta _{1}$ for the three metasurfaces.

### Theory

Consider the bilayer metasurface impinged by a plane sound wave with frequency *f* and at incident angle $\theta$. For wavelengths longer than 2*a* ($\lambda =c/f>2a$), only fundamental modes exist in the ventilation ducts and curled channels, and no diffracted propagating waves are generated by the structure (Fig. 1b). At the left side of the bilayer metasurface, the left-ward (right-ward) propagating wave has an complex amplitude $C_{1}$ ($D_{1}$) in sound pressure. At the right side of the bilayer structure, the complex amplitude of sound pressure is $C_{2}$ ($D_{2}$) for the left-ward (right-ward) propagating wave. Since the sound pressure and particle flow need to be continuous at each structural interface, the field ($C_{2}$, $D_{2}$) at the back (i.e. right) can be related to the field ($C_{1}$, $D_{1}$) at the front (i.e. left) by a $2\times 2$ transfer matrix *M*

2 $$\begin{aligned} \left( \begin{array}{l} C_{2} \\ D_{2} \end{array} \right) =M\left( \begin{array}{l} C_{1} \\ D_{1} \end{array} \right) , \end{aligned}$$

where $M=U_{2}P_{1}P_{2}P_{1}U_{1}$, $$P_{2}=\left( \begin{array}{ll} \exp \left( ikd\right) &\quad 0 \\ 0 &\quad \exp \left( -ikd\right) \end{array} \right)$$ is the transfer matrix for the region between the two metasurfaces, $k=2\pi /\lambda$ is the wavenumber in air, and $P_{1}U_{1}$ and $U_{2}P_{1}$ are the transfer matrices for the front and back metasurfaces. The matrix $P_{1}$ is given by

3 $$\begin{aligned} P_{1}=\left( \begin{array}{ll} q_{1} &\quad q_{2} \\ q_{2} &\quad q_{1} \end{array} \right) \left( \begin{array}{ll} e^{ikb} &\quad 0 \\ 0 &\quad e^{-ikb} \end{array} \right) \left( \begin{array}{ll} q_{3} &\quad q_{4} \\ q_{4} &\quad q_{3} \end{array} \right) , \end{aligned}$$

where $q_{1}=\left( 1+p\right) /2$, $q_{2}=\left( 1-p\right) /2$, $q_{3}=\left( 1+1/p\right) /2$, $q_{4}=\left( 1-1/p\right) /2$, and $p=p_{o}/\cos \theta$. $p_{o}=S_{o}/a^{2}$ is the open area ratio of the metasurface. The matrices $U_{1}$ and $U_{2}$ are given by

4 $$\begin{aligned} U_{j}=\left( \begin{array}{ll} 1-u_{j} &\quad -u_{j} \\ u_{j} &\quad 1+u_{j} \end{array} \right) , \end{aligned}$$

where $u_{j}=p_{c}(1+\beta _{j}i)(1-g_{j})/[2(1+g_{j})\cos \theta ]$, $i^{2}=-1$, $p_{c}=S_{c}/a^{2}$, $g_{j}=\exp \left( 2ik_{cj}L\right)$, $k_{cj}=(1+\beta _{j}i)k$ and $\beta _{j}$ are the wavenumber and sound loss in the curled channel, and the subscript $j=1$ and 2 for the left and right metasurfaces. The sound loss $\beta =0$ is assumed in the ventilation ducts.

Based on the above transfer matrix ($$M\equiv \left( \begin{array}{ll} M_{11} &\quad M_{12} \\ M_{21} &\quad M_{22} \end{array} \right)$$), the reflection *R*, transmission *T*, and absorption *A* of the bilayer metasurface can be obtained. For sound waves incident from the left ( $D_{2}=0$), we have

5 $$\begin{aligned} A=1-R-T, \end{aligned}$$

where $R=\left| M_{21}/M_{22}\right| ^{2}$ and $T=\left| M_{11}-M_{12}M_{21}/M_{22}\right| ^{2}$. Perfect sound absorption can be achieved at resonant frequency ($A=1$ at $f_{R}$) when the losses and spacing are optimized

6 $$\begin{aligned} \beta _{1} &= \beta _{1m}=2S_{c}/\left( \pi a^{2}\cos \theta \right) , \end{aligned}$$

7 $$\begin{aligned} \beta _{2} &= 0, \end{aligned}$$

8 $$\begin{aligned} d\approx & (2n-1)\lambda _{R}/4-2b, \end{aligned}$$

where *n* is a positive integer^18^. We note that when the back metasurface has no absorption loss ($\beta _{2}=0$), it can present complete reflection at resonant frequency $f_{R}$. When the openings of the two labyrinthine resonators in a unit cell have a distance of $(2n-1)\lambda _{R}/4$, the back resonator can cause zero particle velocity at the opening of the front resonator at $f_{R}$. To further obtain complete absorption at $f_{R}$, a critical sound loss ($\beta _{1}=\beta _{1m}$) is required in the front curled channels.

**Figure 4:**
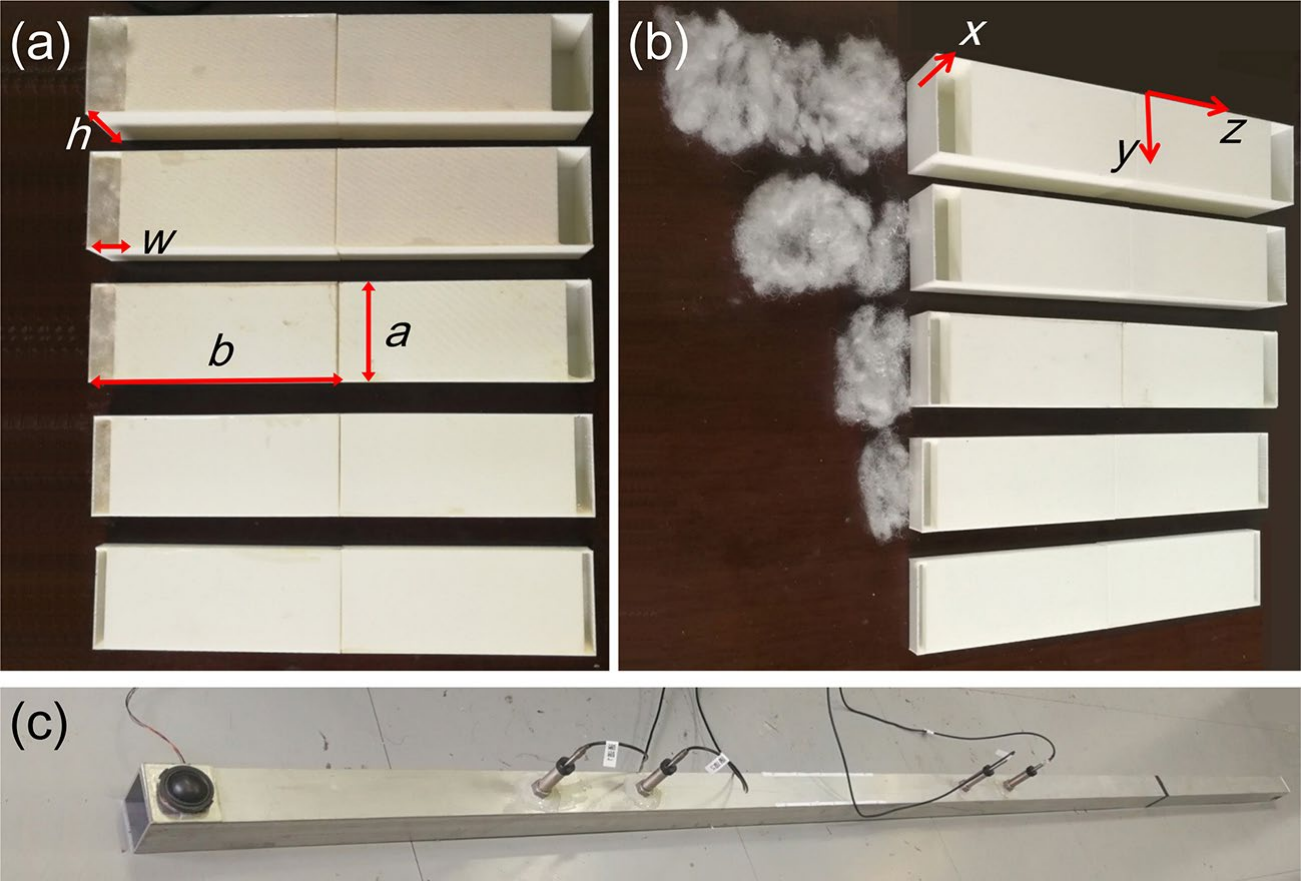
Photographs of experimental samples and setup. (**a**) Five experimental samples. Each sample consists of two boxes with curled, one-end-closed channel inside, where the left box contains appropriate amounts of porous media (polyester pillow stuffing) and the right box is hollow. (**b**) The same as (**a**) but with the porous media taken out from the sample. (**c**) The impedance tube for sound absorption measurements.

**Figure 5:**
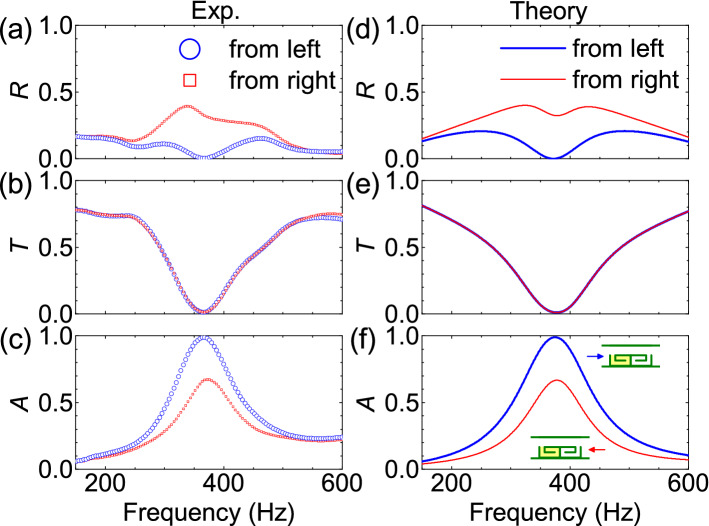
Performance of a 3D printed bilayer labyrinthine metasurface with spacing $d=0$ and open area ratio $p_{o}=0.5$. The left curled channel in the unit cell contains an appropriate amount of porous media, and the right curled channel is hollow. Other parameters are $a=43$ mm, $b=107$ mm, and $t=1$ mm. (**a**)–(**c**) Measured reflection, transmission, and absorption spectra. (**d**)–(**f**) Calculated reflection, transmission, and absorption spectra. Blue and red symbols/curves are for sound waves incident from the left and right, respectively.

### Simulations

To verify the above theory, we perform simulations for bilayer ventilated labyrinthine metasurfaces, which have $h=0.5a$, $t\ll a$, and thus an open area ratio $p_{o}=0.5$. Sound waves are incident normally from the front ($\theta =0$). We first consider the case with a zero spacing between the two metasurfaces ($d=0$). In Fig. 2a, curve I shows the calculated absorption spectrum with losses of $\beta _{1}=0.161$ and $\beta _{2}=0$ in the front and back metasurfaces. A maximal absorption ($A_{R}=99.5\%$) can be seen at resonant frequency ($f_{R}=0.247c/L$). The absorption at resonance $A_{R}$ depends on the losses in the curled channels as shown in Fig. 2b. Equi-absorption contours are also plotted for different absorptions ($A_{R}=0.90$, 0.94, 0.98) in Fig. 2b. We see that the absorption $A_{R}$ approach a maximum $A_{Rm}$ (99.5%) at $\beta _{1}=\beta _{1m}=0.161$ and $\beta _{2}=\beta _{2m}=0$, agreeing well with analytic results from Eqs. (6) and (7) ($\beta _{1m}=0.159$ and $\beta _{2m}=0$). We note that $A_{R}$ remains high around the optimal losses ($\beta _{1m}$, $\beta _{2m}$). If the front and back metasurfaces possess the same optimal losses ($\beta _{1}=\beta _{2}=0.148$) in the curled channels, the maximal absorption can still be as high as 95% (see Fig. 3). But if only the front metasurface exists, the maximal absorption will decrease to 71% even with using an optimal loss ($\beta _{1}=0.108$) (see Fig. 3).

More results are shown in Fig. 2c for bilayer metasurfaces with different spacing *d*. It is found that the maximal absorption $A_{Rm}$ varies with increasing the spacing *d*. Near perfect absorption ($A_{Rm}>99\%$) can be achieved in some ranges including $0<d<0.24L$ and $1.94L<d<2.24L$. But if the spacing is not in such optimized regions, low absorption will occur. For instance, at $d=0.66L$ and even with using optimal losses ($\beta _{1m}=0.321$, $\beta _{2m}=0.115$), the absorption $A_{Rm}$ can be only 59.3%, indicating the importance of the spacing *d* for achieving perfect absorption. The corresponding absorption spectrum is plotted as curve II in Fig. 2a, where two wide resonant peaks are visible at frequencies of $0.208c/L$ and 0.281*c*/*L*. It should also be mentioned that a very sharp absorption peak can occur at a critical spacing ($d=0.7L$) with tiny optimal losses of $\beta _{1m}=0.0073$ and $\beta _{2m}=0$ (see curve III in Fig. 2c). We note that such a high-*Q* resonant mode can be viewed as an acoustic quasi bound state in the continuum (BIC), which can exist in various acoustic systems^43^.

To clarify the above results, the distribution of particle velocity in a unit cell of the bilayer metasurface is simulated at resonant frequency using a finite-element method (COMSOL Multiphysics), as shown in Fig. 2d. When $d=0$, the openings of the two labyrinthine resonators in the unit cell have a distance of about a quarter of resonant wavelength ($\sim \lambda _{R}/4$). Since a large particle velocity occurs at the opening of the back resonator, zero velocity can be obtained at the opening of the front resonator (see case I in Figs. 1d and 2d). Therefore, a single absorption peak is visible (curve I in Fig. 2a) and its strength can be 100% with using an appropriate loss in the front resonator. In contrast, if the spacing between the two metasurfaces is not appropriately chosen, the ventilation duct can also serve as a resonator but with a zero loss ($\beta =0$) (see case II in Figs. 1d and 2d). Hence, two resonant peaks occur with imperfect strengths (see curve II in Fig. 2a).

### Experiments

Based on the above theoretical results, we fabricated bilayer ventilated labyrinthine metasurfaces with polylactic acid (PLA) by means of 3D-printing technology (see Fig. 4a and b and Methods). The structural parameters are $a=43$ mm, $b=107$ mm, $t=1$ mm, $d=0$, and $p_{o}=0.5$. By using an impedance tube with a square cross section (see Figs. 1c, 4c, and Methods), the reflection, transmission, and absorption spectra were measured for a unit cell, as shown in Fig. 5a–c. For the unit cell, the back resonator is hollow whereas the front one contains an appropriate amount of porous media (with a mass of 0.1498 g; see Methods). It is found that for sound waves incident from the front, the unit cell exhibits a low reflection (17%) in a wide frequency range (100-800 Hz). Very low reflection ($R=0.2\%$), low transmission ($T=1.2\%$), and near-perfect absorption ($A=98.6\%$) can be seen at resonant frequency ($f_{R}=368$ Hz). For sounds incident from the back, a lower absorption (67%) is found at resonant frequency due to the asymmetric loss of the unit cell ($\beta _{1}>\beta _{2}$). It should also be mentioned that almost the same transmission is observed for sounds from the front and back, agreeing with the theoretical expectation.

Numerical calculations were conducted for the above sample based on the transfer-matrix method (Fig. 5d–f). In the calculations, a thin layer of air (thickness = 0.6 mm) is considered to be static at the surface of each wall. We calculated absorption spectra for different losses ($\beta _{1}$, $\beta _{2}$). It is found that when $\beta _{1}=0.11$ and $\beta _{2}=0.03$ are used in the front and back curled channels, the strength and width of the calculated absorption peak can match the experimental values. Since slight sound loss ($\beta _{2}=0.03$) exists in the back channel, the back resonator cannot provide complete reflection. Hence, for sound incident from the back, a considerable absorption ($A_{back}=67\%$) is observed in experiments. If an ideal asymmetric absorption (i.e. $A_{back}=0$ and $A_{front}=100\%$) is desired, the back channel should exhibit zero sound loss ($\beta _{2}=0$).

**Figure 6:**
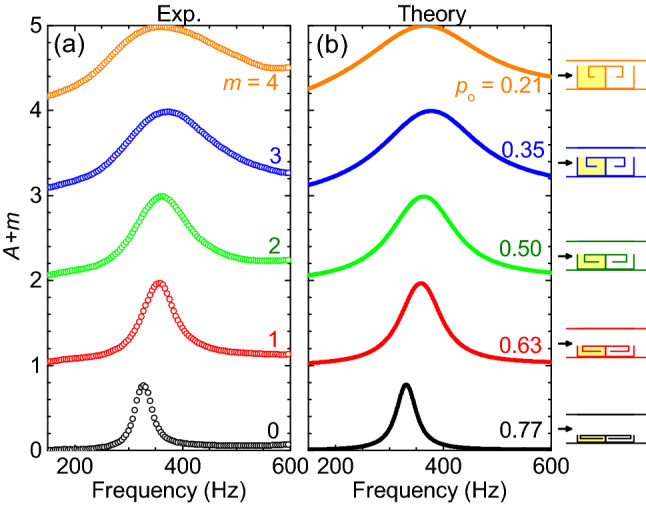
Absorption of 3D printed acoustic bilayer metasurfaces with spacing $d=0$ and different open area ratios $p_{o}$. The left curled channel in the unit cell contains an appropriate amount of porous media, and the right curled channel is hollow. Other parameters are the same as those in Fig. 3. (**a**) Measured and (**b**) simulated absorption spectra. Sound waves are incident from the left.

**Figure 7:**
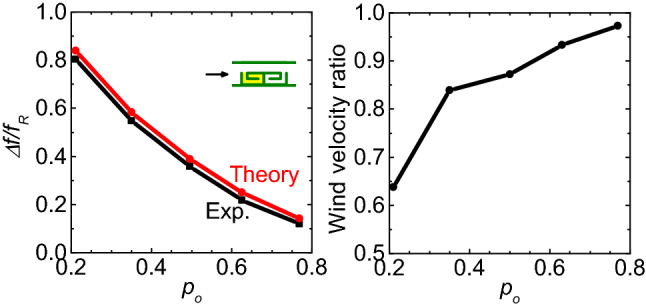
(**a**) Relative absorption bandwidths $\Delta f/f_{R}$ and (**b**) measured wind velocity ratio as a function of open area ratio $p_{o}$.

Besides the absorption strength, the absorption bandwidth is also important for a sound absorber. For the above sample, the measured absorption is higher than 50% for frequencies ranging from 305 Hz to 435 Hz, corresponding to a relative band width $\Delta _{f}/f_{R}$= 36% with the full width at half maximum $\Delta _{f}=129.4$ Hz. We note that the thickness of the bilayer metasurface is in a subwavelength scale (λ/5h) compared to the lower limit (305 Hz) of the absorption band. In addition, the relative bandwidth of absorption here is much larger than (7 times of) recent results with using a pair of Helmholtz resonators in the unit cell^37^. If the same wide absorption band is desired, seven pairs of Helmholtz resonators need to be adopted in the unit cell^37^. Moreover, when more different resonators are adopted in the unit cell, their coupling effects become more complex, resulting in lower peak absorption ($<92\%$) observed in experiments^37^.

The absorption bandwidth can be further tuned by adjusting the open area ratio of the bilayer metasurface, as shown in Figs. 6a and 7a. Here, more unit cells were fabricated with open area ratios being 0.21, 0.35, 0.63, and 0.77, of which measured relative absorption bandwidths are 80%, 55%, 22%, and 12%. For the four unit cells, the front resonators contain appropriate amounts of porous media (with masses of 0.6212 g, 0.3644 g, 0.0563 g, and 0 g) while the back resonator remains hollow. To reproduce the experimental results with simulations, the losses ($\beta _{1}$, $\beta _{2}$) are set as (0.184, 0.03), (0.146, 0.03), (0.08, 0.03), and (0.045, 0.045) for the four samples, respectively. We can see that the absorption band becomes wider with decreasing the open area ratio of the structure. The simulated results agree well with the experimental observation (see Figs. 6 and 7a).

The ventilation performance is further characterized for the bilayer labyrinthine metasurfaces. Here, a unit cell of the metasurface is placed in an aluminum tube with a square cross section of a size 44 mm. One end of the tube is seamlessly connected with the outlet pipe of an electric blower (9028E, Anjieshun, China). An anemometer (DP-1000-IIIB, Yiou, China) is used to monitor the air flow velocity at a fixed position in the tube. When the tube is hollow, the wind velocity is $v_{wo}$ (8 and 15 m/s were tested in our experiments). When the unit cell is placed in the tube, the wind velocity becomes $v_{w}$. Thus, a ventilation rate can be defined as the ratio of wind velocity ($v_{wo}/v_{w}$)^27,37^. Figure 7b shows the measured wind velocity ratio for different unit cells. We can see that the wind velocity ratio increases with increasing the open area ratio of the structure. For the above five samples, the ventilation rates are larger than 0.6.

## Discussion

In summary, we design, fabricate, and characterize a bilayer ventilated labyrinthine metasurface for perfect sound absorption and free air flow. Both a ventilation duct and a curled single-port channel exist in the constituent single-layer metasurface. By using asymmetric losses in the two single-layer metasurfaces and adjusting their spacing, perfect sound absorption is achieved at resonant frequency for sound waves incident from the front. The measured peak absorption is as high as 98.6% even at a relatively large open area ratio (50%). By tuning the open area ratio, the relative absorption bandwidth can be adjusted in a large range (from 12% to 98%). For sounds incident from the back, the bilayer metasurface still serves as a sound barrier with low transmission and partial reflection/absorption. Our work provides a strategy for achieving broadband perfect sound absorption in ventilated structures and could be extended to other fields such as electromagnetic waves and water waves.

## Methods

### Sample preparations

Each experimental sample is composed of two boxes, and each box contains a labyrinthine structure (see. Fig. 1a) and a side plate. The two parts are first fabricated with PLA by 3D-printing technology, then agglutinated together. The porous media placed in the box is polyester pillow stuffing (white 15d$\times$51mm hcs 100% polyester stuffing pp staple fiber filling pillow).

### Sound absorption measurements

A commercial impedance tube (Hangzhou Aihua, AWA6290T) was applied to measure the absorption of acoustic metasurfaces (see Figs. 1c and 4c). Here, a unit cell of the bilayer metasurface was placed in an aluminum impedance tube, which has a total length of 2.9 m, a wall thickness of 3 mm, and a square cross section with an inner size of 44 mm. A loudspeaker is placed at the left end of the impedance tube. The right part of the impedance tube is filled with sound-absorbing materials of a length of 1.4 m, so that the resulted reflection can be less than 1.5% in the frequency band of interest ($f>250$ Hz). Four 1/4-in. condensed microphones are situated at designated positions to sense local pressure. The loudspeaker was fed with a sinusoidal signal of which the frequency increases with increasing time. By analyzing the signals from microphones, the reflection, transmission, and absorption spectra can be obtained for the unit cell.
